# Preliminary Evidence of Feasibility, Acceptability, and Potential Benefit of a Culturally Responsive Mental Health Engagement Program for Hispanic Adults, *Pensamientos y Pláticas*

**DOI:** 10.3390/healthcare14152240

**Published:** 2026-07-23

**Authors:** Jason Mallonee, Rosa Escalante, Eden Hernandez Robles, Karen Kwon, Brittany Ochoa, Monica Smith, Vanessa Medrano, Emre Umucu

**Affiliations:** 1 Department of Social Work, College of Health Sciences, The University of Texas at El Paso, 500 W. University Ave., El Paso, TX 79968, USA; ehrobles@ollusa.edu (E.H.R.); kkwonmanriquez@gmail.com (K.K.); brittany.ochoa44@gmail.com (B.O.); thesmithmonica@gmail.com (M.S.); vlmedrano915@gmail.com (V.M.); 2 Interdisciplinary Health Sciences, The University of Texas at El Paso, 500 W. University Ave, El Paso, TX 79968, USA; reescalante@miners.utep.edu; 3 Worden School of Social Service, Our Lady of the Lake University, 411 SW 24th St., San Antonio, TX 78207, USA; 4 Department of Public Health Sciences, The University of Texas at El Paso, 500 W. University Ave., El Paso, TX 79968, USA; eumucu@utep.edu; 5 College of Health Sciences, Research, Evaluation, and Academic Center on Health Disparities, The University of Texas at El Paso, 500 W. University Ave, El Paso, TX 79968, USA; 6 Institute for Health and Lifespan Research, The University of Texas at El Paso, 500 W. University Ave, El Paso, TX 79968, USA; 7 Audie L. Murphy Memorial VA Hospital, South Texas VA Medical Center, 7400 Merton Minter, San Antonio, TX 78229, USA

**Keywords:** mental health, stigma, interventions, Hispanic, Latino, cultural responsiveness

## Abstract

**Background/Objectives:** Hispanic adults in the United States seek and access professional mental health services at a disproportionately lower rate than non-Hispanic White adults, despite having similar rates of mental health challenges. Community focus groups held in El Paso, Texas, in 2022 revealed both systemic and individual-level factors that serve as barriers to help-seeking and access. Participant recommendations for overcoming these barriers included increasing mental health education and improving the cultural responsiveness and navigability of mental health services. Stemming from these findings and informed by the literature on trauma-informed practice, culturally responsive interventions, and evidence-informed practices, we developed and pilot-tested a 4-week closed group community-grounded mental health engagement program called *Pensamientos y Pláticas*. **Methods:** The program was offered to 82 participants. Paired-sample *t*-tests were used to analyze differences between pre- and post-intervention data for the outcome variables assessed: stigma and emotional well-being. Analysis was limited to the 39 participants who completed at least half of the modules and had intact pre- and post-intervention data. **Results:** Data analysis revealed a statistically significant reduction in stigma related to help-seeking with a small-to-moderate effect size, while no statistically significant increase in emotional well-being was supported by the data. **Conclusions:** Findings from this pilot study reflect preliminary evidence of feasibility, acceptability, and potential benefit at reducing stigma related to seeking help in Hispanic communities and support *Pensamientos y Pláticas* as a promising culturally responsive stigma-reduction intervention.

## 1. Introduction

Hispanic adults in the United States experience similar rates of mental health conditions when compared to non-Hispanic White adults. In 2023, 20.6% of Hispanic adults reported experiencing mental health conditions compared to 23.2% of non-Hispanic White adults [1]. Despite the similarity in prevalence of mental health conditions among these two populations, only 47.4% of Hispanic adults reported receiving mental health treatment for those conditions in 2023, compared to 58.7% of non-Hispanic White adults [1]. The mental health disparities experienced by Hispanic adults, consistently reflected in national data over time, suggest that current efforts are not significantly reducing these mental health disparities. The persistence of these disparities is particularly salient given that Hispanics are the fastest-growing ethnic minority group in the country, reaching 68 million in 2024 and reflecting one in five Americans [2]. Addressing this disparity through developing community-grounded, culturally responsive, and linguistically appropriate interventions may be a promising solution.

A clear understanding of the barriers that Hispanic adults experience in seeking and receiving mental healthcare is essential for informing intervention strategies. Mental health treatment for Hispanic adults is characterized by less frequent treatment utilization, shorter episodes of care, inadequate care, and fewer therapy referrals [3]. Systemic and structural factors that serve as barriers to help-seeking and treatment utilization for Hispanic populations include poverty, lack of health insurance, transportation challenges, lack of childcare, and a lack of linguistic congruence between providers and clients [4,5,6]. Even when providers speak Spanish, there is often a lack of bilingual materials available to those practitioners [7]. Stigma-related factors like cultural beliefs and shame around mental health further reduce the likelihood of Hispanic adults seeking and engaging in mental health treatment [8,9,10].

An additional challenge related to engaging Hispanic adults in mental health services is a shortage of culturally responsive interventions. Culturally responsive interventions for Hispanic populations account for the unique contexts and treatment needs that this community experiences [11], and these interventions are shown to improve mental health outcomes [12,13]. Whether due to systemic barriers or stigma-related barriers, less frequent mental health service utilization creates a higher potential for unresolved mental health conditions for this population. Unresolved mental health conditions are associated with a range of negative outcomes, including poorer physical health, higher rates of unemployment, and overrepresentation among the unhoused and incarcerated [14,15,16]. Given the detrimental outcomes associated with mental health conditions, there is a critical need to reduce barriers to seeking and receiving mental healthcare for those currently underserved by existing service delivery systems through the development and implementation of more diverse and community-grounded strategies.

Community-based and outreach programs aimed at increasing access to mental health services within Hispanic communities have demonstrated greater effectiveness than merely targeting one barrier, such as linguistic incongruence [17]. The demonstrated effectiveness of community-based and outreach programs highlights the need to directly engage the communities the intervention intends to serve in the program development process. Evidence points toward engaging with Hispanic community members within their cultural and community contexts [17,18] as a more effective strategy for reducing mental health disparities.

Taking these factors into consideration, the research team set out to ascertain barriers experienced by Hispanic community members in the Paso del Norte U.S.–Mexico border region and to elicit recommendations from community members on how to increase mental health equity. To accomplish these goals, the research team conducted four focus groups with a total of 25 Hispanic community members. Study participants were recruited from a local comprehensive food pantry, and the focus groups were held at this setting. Given that for Hispanic adults, lower socioeconomic status and an inability to meet basic needs are associated with poorer mental health outcomes, less frequent patterns of help-seeking, and lower rates of mental health utilization [19,20,21,22,23], the research team determined that recruiting from this setting would likely result in richer and more authentic perspectives from those more likely to be impacted by mental health challenges. Meeting focus group participants in this community setting, rather than in a community mental health clinic, was thought to increase the likelihood of attracting participants for whom mental health stigma surrounding treatment settings may have been a barrier to participation in the focus groups.

Common themes emerging from the focus groups included shame as a barrier to help-seeking and treatment utilization, as well as individual factors (e.g., a lack of time, living in poverty, a lack of experienced progress when having used treatment, lack of family support, and a lack of knowledge about mental health) [24]. Shared systemic challenges included lack of insurance or financial means, provider shortage and high turnover, longer wait times, and broken referral systems and processes [24]. Recommendations for agencies, providers, and researchers included increasing outreach and education efforts, improving service delivery systems, promoting person-centered processes, and continuing to engage the community in research and dialogue [24].

Taking into account the existing literature and findings from these focus groups, the research team developed an innovative, community-grounded, culturally responsive, and linguistically appropriate mental health engagement intervention. With regular input and assistance from members of a Community Advisory Board consisting of agency leaders, mental health providers, and people with lived experience of mental health conditions, *Pensamientos y Pláticas* was established. The following sections summarize the pillars undergirding the program, the program’s components and structure, and the strategies the research team employed to assess the program’s feasibility, acceptability, and potential benefit.

## 2. Overview of *Pensamientos y Pláticas* Mental Health Engagement Intervention

*Pensamientos y Pláticas* grew out of community focus group recommendations for reducing regional barriers to seeking and accessing mental health services, including providing education on mental health conditions and increasing community engagement and outreach [24]. The program incorporates strategies known to increase mental health treatment engagement more broadly by integrating mental health programming into settings where the primary focus is not on treating mental health conditions and by using it as a complement to programs targeting other needs [25,26]. *Pensamientos y Pláticas* represents a new pathway for engaging community members in conversations about mental health and facilitating access to mental health services. The development of this educational and skill-building group community intervention was guided by the following four pillars:

### 2.1. The Four Program Pillars

**Pillar One: Community Engagement.** The development of *Pensamientos y Pláticas* began with engaging community members to learn how they viewed mental health and perceived barriers to seeking and accessing mental healthcare, specifically in the Paso del Norte region of the U.S.–Mexico border. In addition to holding community focus groups, the research team formed a Community Advisory Board (CAB) consisting of mental health providers and agency leaders, those with lived experience of mental health conditions, and other community stakeholders. The CAB oversaw program development and provided invaluable feedback that is reflected in the finalized program components. The research team took this “bottom-up” approach because evidence-based practices alone often fail to represent the unique needs of communities of color [27]. By rooting the program development process in the community, *Pensamientos y Pláticas* reflects the high value placed on communities as experts of their own experiences and needs, and whose contributions to providing the evidence to inform programs are as valuable as those established through the evidence-based practice movement. Centering community expertise results in a program that is community-grounded and evidence-informed.

Some examples of incorporating community-generated evidence from the research team’s qualitative study [24] into *Pensamientos y Pláticas* include: (1) supporting the expression of a variety of beliefs and viewpoints; (2) facilitating opportunities for sharing of personal experiences, normalizing conversations around mental health; (3) providing education on mental health conditions; (4) addressing individual and system-level barriers; (5) strengthening coping strategies, including the use of natural supports and non-professionalized methods for managing mental health; and (6) engaging participants as experts of their own experience.

**Pillar Two: Cultural Responsiveness.** In addition to engaging community members to ensure cultural responsiveness, the intervention also incorporated other evidence-based strategies. Utilizing Bernal et al.’s seminal framework [28] ensured cultural responsiveness of approaches to mental health interventions. Evidence of incorporating Bernal et al.’s framework into *Pensamientos y Pláticas* include: (1) offering the program in participants’ preferred language, either Spanish or English; whenever possible, selecting facilitators with similar cultural and linguistic backgrounds as participants; (2) guiding facilitators through a process of recognizing their own positionality and how it may impact program facilitation; (3) incorporating culturally relevant language, terminology, values, and customs by positioning participants as experts of their own experiences and facilitating meaningful reflection and sharing; and (4) fostering participant connection and community building through storytelling and valuing personal narratives (rooted in concepts of *cuentos* and *testimonios*), which is congruent to the context and culture of participants.

**Pillar Three: Trauma-Informed Care.** The purpose of trauma-informed care is to provide programs in a way that is accessible and appropriate for those who may have experienced trauma by employing effective trauma-informed approaches to practice [29,30,31,32]. Incorporating principles of trauma-informed care into *Pensamientos y Pláticas* program development included: (1) ensuring physical and emotional safety by facilitating welcoming, validating, and affirming experiences for participants; (2) emphasizing choice in how participants engage with the program; (3) engaging with participants in a collaborative process rooted in the guiding principles of respect, empathy, confidentiality, wisdom, and accountability; (4) fostering trust through guiding principles and thorough training of facilitators; and (5) empowering participants through focusing on their strengths and increasing coping skills and supports.

**Pillar Four: Evidence-Informed Practice.** While *Pensamientos y Pláticas* is not intended to treat any mental health condition, incorporating evidence-based practice can strengthen mental health engagement. The approaches taken in each of the program’s modules are rooted in a variety of evidence-based frameworks, including person-centered, narrative, and solution-focused approaches; motivational interviewing; mental health awareness and literacy; and systems navigation. Recognizing that mental health treatment disparities for Hispanic populations have remained largely unchanged throughout time, this intervention represents an effort to introduce a novel and innovative strategy that adds to the existing evidence base supporting culturally responsive interventions.

### 2.2. The Program Modules

**Module One: My Story.** The goal of module one is for participants to learn more about *Pensamientos y Pláticas*, establish trust and rapport, and explore their own lives through storytelling. *Cuentos*, or storytelling, is a particularly valuable tool for building community and reducing mental health stigma by honoring cultural norms and reducing fears around discussing mental health [33]. The storytelling (*cuentos*) approach helps participants build cultural resilience and may support them in seeking a healing process [34]. The intention behind grounding the program in storytelling is to foster connection, lay a foundation of trust, and promote authentic, open engagement in what can be difficult conversations about mental health throughout the program. This first module begins with introductions, a review of the program and module, and establishing an understanding of the guiding principles from participants’ perspectives. The storytelling process begins with the introduction of the Participant Workbook, in which participants complete and reflect upon activities throughout the program. In this module, participants reflect on their decision to join the group; initial thoughts on mental health; the challenges they have faced, the support they have received, and how they have coped; and the role of culture in their lives. Participants share aspects of their stories as they feel comfortable, and the group reflects on the storytelling process. The module ends with a final reflection and a reminder to bring 2–3 objects or pictures that represent what’s most important to them to their next meeting.

**Module Two: What is Mental Health? How Do I Separate Fact from Fiction?** The goal of module two is for participants to learn more about mental health; explore how they view mental health, wellness, and mental health conditions; and engage in activities to promote positive thinking. In module two, participants begin targeting the impact of stigma on the decision to discuss mental health and/or seek out services. In this module, stigma is conceptualized as consisting of potential individual biases towards mental health and treatment, cultural stigma, and disparities related to access to information and treatments [35,36]. A central focus of this module is cultural beliefs, as programs that incorporate cultural responsiveness are more likely to reduce stigma and encourage help-seeking and treatment utilization [37,38]. To begin this module, participants engage in a check-in and reintroduction activity in which they share the importance of the 2–3 pictures or objects they brought or describe the items they would have brought. Following the group’s review of the guiding principles, the module purpose is reviewed to ensure shared understanding. A series of prompts are utilized to elicit reflections on mental health and wellness, mental health conditions, and cultural perceptions of mental health. Psychoeducation is provided to cover mental health and wellness, mental health conditions, both general and specific conditions, and coping strategies. For each prompt, participants reflect in their Participant Workbook and can choose to share with the larger group at different points during the activity. To close the module, participants engage in a final reflection on changes in perceptions of mental health during the session, and receive a reminder to bring a baby picture (printed or on their phone), if possible, to module three.

**Module Three: Building Strengths and Supports.** The goal of module three is for participants to identify their strengths, understand that they deserve support and care, and explore various social support systems in their lives. Drawing upon a strengths-based approach shown to increase empowerment and life satisfaction [39,40], this module is anchored in research demonstrating the protective effects of social support systems on mental health outcomes across a range of physical and mental health conditions [41,42,43]. The third module begins with a check-in, a review of the guiding principles as understood by the group, and a review of the module’s purpose. Following a community-building icebreaker, participants reflect on and share their perceptions of support systems, then complete a support system map in their Participant Workbook. By engaging in this activity, participants can brainstorm existing supports and identify others who may be a source of support in their lives. The following two activities are designed to build strengths and empower participants. The first activity involves reflecting on the changes and growth experienced throughout their lives, using the baby pictures brought to the session as a frame of reference. In the second activity, participants take a $20 bill in good shape and pass it around the group for others to crinkle, fold, etc. The bill is then flattened, and participants reflect upon how it is still worth the same amount despite the wear and tear. These activities reinforce growth and transformation and emphasize that one’s value does not change over time, even after experiencing hardship. At the end of the module, participants engage in a final reflection on their experiences with these activities.

**Module Four: Creating a Plan.** The goal of module four is to support participants in reflecting on their accomplishments and successes, developing an individualized plan for caring for their mental health, and learning how to navigate and use the Mental Health Resource Guide at the end of the Participant Workbook. The celebration of participants’ accomplishments, coupled with planning for continued mental health support, forms the central focus of this module. Recognizing that termination of any shared experience can cause sadness and distress [44], focusing on future goals helps support healthy group closure [45]. Like other modules, this module begins with a check-in, a review of the guiding principles as understood by the group, and a review of the module’s purpose. Participants are guided through a series of reflection prompts that explore what they have learned in the program, changes in thinking about mental health, a review of strengths and support systems, and the development of a coping toolbox. As throughout the program, participants first reflect in their Participant Workbook and then share as little or as much with the group as they feel comfortable. The group then role-plays certain scenarios and how they can take care of their mental health. After a review of the Mental Health Toolbox (which includes reminders of what was learned and possible resources), facilitators provide one-on-one support for navigating resources, including providing direct navigational assistance after or shortly after completion of the program if requested by a participant.

## 3. Materials and Methods

The four modules of *Pensamientos y Pláticas* are designed to be delivered face-to-face with a closed group format over 4 weeks (once a week for 90 min each session). For this exploratory pilot study, the intervention was delivered in six community-based settings where the agency’s primary purpose was not mental health treatment. These included a food pantry, a shelter for those who have experienced violence, a shelter for the unhoused, a community center, an organized group of new mothers, and a group of community health workers and *promotores*. The program was brought to the agencies to reduce transportation barriers participants may have experienced and to provide it in a familiar environment. The intervention was delivered from March through November 2024. Data were collected pre- and post-intervention to collect sociodemographic information and to assess the program’s impact on mental health stigma and emotional well-being.

### 3.1. Intervention Fidelity

A total of nine graduate and undergraduate social work students completed the facilitator training. The students were selected from the university where the research was conducted and from additional community sites hosting social work field placements. The program developers built a facilitator manual detailing how to facilitate each module step by step. Each facilitator reviewed the manual prior to beginning facilitator training. The facilitators were trained through a two-phase process. In the first phase, program developers facilitated the program to the facilitators. In the second phase, adherence to the module structure was monitored through having the facilitators facilitate the program back to the program developers. Fidelity checks were conducted throughout the study by observing the facilitators at all sites. The program developers conducted regular debriefing and discussion with the facilitators to ensure the intervention was implemented consistently across sites.

### 3.2. Recruitment

After obtaining approval from the Institutional Review Board at The University of Texas at El Paso, recruitment flyers in both Spanish and English were disseminated to various nonprofit organizations throughout the region. Participants were recruited through convenience sampling on an ongoing basis from March through November 2024 for this phase of the exploratory pilot study. Agency staff referred interested participants to the research team for screening. Additionally, some participants contacted members of the research team directly using the team members’ contact information on the recruitment flyers. Inclusion criteria consisted of being at least 18 years old and identifying as Hispanic or Latino. Exclusion criteria included active risk of harm to themselves or others, or disorientation to person, place, time, and/or circumstances. Protocols were in place to route potential participants excluded for these reasons to an immediate mental health evaluation, although no participants screened were excluded for these criteria. Once potential participants were screened for eligibility, the study was explained to them through the informed consent process, and if they met the inclusion criteria and agreed to participate, they were enrolled. Participants were reminded that they could discontinue participation in the study at any time. All recruitment, informed consent, and program materials were available in either Spanish or English.

### 3.3. Study Participants

The research team developed a sociodemographic questionnaire to collect descriptive data for the sample with participants answering questions related to gender, age, race, ethnicity, marital status, education, income, and geographic location. Participants also answered questions related to previous experiences of mental health treatment or mutual aid groups. Participants could choose to answer or not answer any items on this questionnaire. These sociodemographic data were used solely to describe the study’s sample.

Of the 82 participants who enrolled in the pilot study, 39 participants completed at least two of the four modules and had complete pre- and post-intervention outcome data; these participants comprised the analytic sample. Because the analytic sample represented 47.6% of enrolled participants, findings may be affected by attrition and selection bias and should be interpreted as preliminary. Given the repetition throughout the program, the research team determined that attending at least half of the modules and having intact pre- and post-test data would be sufficient to measure the program’s initial impact on outcome variables. Only 39 individuals were included in the data analysis.

## 4. Measures

### 4.1. Mental Health Stigma

In the present study, mental health stigma was measured using the Self Stigma of Seeking Help (SSOSH) scale [46]. The SSOSH questionnaire has been found to have solid psychometric properties, including strong reliability and construct, criterion, and predictive validity [46], as well as cross-cultural validity [47]. Psychometric properties of the SSOSH remained strong when translated into Spanish and used with Spanish-speaking populations specifically [48,49]. The SSOSH scale consists of 10 prompts related to perceived impacts of seeking help for mental health conditions in which participants rate their level of agreement on a 1–5 Likert-type scale (1 = strongly disagree, 2 = disagree, 3 = agree and disagree equally, 4 = agree, and 5 = strongly agree). Example prompts include “I would feel inadequate if I went to a therapist for psychological help” and “My self-esteem would increase if I talked to a therapist.” Half of the items are reverse scored, and then all items are averaged to determine a mean score for each participant, with a higher score associated with a higher level of self-stigma. A mean score across study participants was used for analysis.

### 4.2. Emotional Well-Being

In the present study, emotional well-being was measured using the Warwick Edinburg Mental Well-Being Scale (WEMWBS) [50,51]. The WEMWBS has been found to have strong psychometric properties across multiple studies [50,51] which persisted when validated on Spanish-speaking populations [52]. The WEMWBS is a 14-item questionnaire in which participants report the frequency of certain feelings or experiences over the previous 2 weeks using a 1–5 Likert-type scale (1 = none of the time, 2 = rarely, 3 = some of the time, 4 = often, and 5 = all of the time). Example prompts include “I’ve been feeling optimistic about the future” and “I’ve been feeling good about myself.” Total individual scores could range from 14 to 70 with the higher the score indicating a higher level of emotional well-being. A mean score across study participants was used for analysis.

## 5. Data Analysis

Descriptive statistics were utilized to identify frequency, mean, and distribution of sociodemographic variables. For the two outcome variables, mental health stigma and emotional well-being, prior to data analysis, the data were checked for missing values, and cases with incomplete data were excluded from the final analysis. Descriptive statistics were calculated for all study variables. Participants with any missing pre- or post-intervention outcome data were excluded from the paired-samples analyses. Paired-samples *t*-tests examined differences in WEMWBS total scores and SSOSH scores between pre-test and post-test. Finally, effect sizes were calculated using Cohen’s d and Hedges’ g to evaluate the magnitude of observed changes, with Hedges’ g applied as a bias correction recommended for small sample sizes. A 95% confidence interval (CI) was used for all statistical tests, with significance set at *p* < 0.05.

## 6. Results

### 6.1. Participant Characteristics

Of the 39 participants included in the analysis, all identified as Hispanic or Latino through the screening process and self-described their race and ethnicity as follows: Hispana (9), Latina (5), Mexicana (5), Hispanic (3), Hispano (3), Black (2), Blanca (2), Black/Mexican/Italian (1), Cuban/American (1), Hispana/Latina (1), Hispana/Mexicana (1), Latina/Boliviana (1), and Latina/Hispana (1) with four participants choosing not to further specify their race and ethnicity. The overwhelming majority identified as female (38), with one of these female-identified participants also self-describing their gender as non-binary. One participant chose not to disclose their gender identity. Participant ages ranged from 24 to 78 years, with a mean age of 50.6 (SD = 12.9). In terms of marital status, participants identified as follows: single (13), married (12), separated (4), divorced (4), widowed (3), and other (2), with one participant choosing not to disclose marital status.

Education levels varied with participants reporting the following levels of education: no schooling (2), elementary school (5), middle school (3), high school (4), high school general educational development (GED) or other high school equivalent (6), technical college (4), some college (2), bachelor’s degree (8), and master’s degree (2) with three participants choosing not to disclose their level of education. Annual household income varied, with participants reporting the following: $0 (8), $1–$9999 (6), $10,000–$24,999 (11), $25,000–$49,999 (5), and $50,000–$74,999 (1), with eight participants choosing not to disclose income levels. Notably, over two-thirds of participants reported annual incomes below $25,000.

In terms of previous experiences with mental health, 12 participants reported previous use of mutual aid/self-help groups compared to those who did not (21) and those who chose not to disclose (6). Most participants reported a perceived need for mental health services (20) compared to those who did not (13) and those who chose not to disclose (6). Slightly below half of participants reported previous use of mental health services (18) compared to those who did not (15) and those who chose not to disclose (6). All participants who had previous experience with mental health services reported receiving counseling and/or psychiatric services. See Table S1 for full details of sociodemographic variables.

### 6.2. Intervention Analysis

The mean WEMWBS total score at pre-test was 52.41 (SD = 11.50), while the mean post-test score was 55.79 (SD = 11.34). Results of the paired-samples *t*-test for WEMWBS total scores indicated that the increase from pre-test to post-test was not statistically significant, t(38) = −1.94, *p* = 0.060, 95% CI for the mean difference [−6.91, 0.15], Cohen’s d = −0.31, Hedges’ g = −0.30. In contrast, the mean SSOSH score at pre-test was 3.04 (SD = 0.86), and the mean post-test score was 2.71 (SD = 0.81). Results of the paired-samples *t*-test for SSOSH scores revealed a statistically significant decrease, t(38) = 2.22, *p* = 0.032, 95% CI for the mean difference [0.03, 0.63], Cohen’s d = 0.36, Hedges’ g = 0.35. The effect size for SSOSH scores was small-to-moderate (Cohen’s d = 0.36; Hedges’ g = 0.35), suggesting a promising preliminary reduction in self-stigma related to seeking psychological help among participants retained in the analytic sample. See Table S2 for full statistical analysis.

## 7. Discussion

The findings presented here reinforce a well-documented need to better understand the mental health needs and barriers to care experienced by the Hispanic populations in the United States [53]. Cultural responsiveness remains central to effective intervention design for Hispanic populations, as cultural norms and expectations often shape accessibility, utility, and acceptability [53]. Indeed, integrating cultural components in working with Hispanic populations is more likely to reduce mental health treatment disparities [53]. In contrast to dominant approaches to mental health service delivery that rely solely on the Spanish translations of intervention manuals and materials, *Pensamientos y Pláticas’* culturally grounded framework emphasizes community values and knowledge.

The development of *Pensamientos y Pláticas* reflects a community-grounded, culturally responsive, and linguistically appropriate effort to create an educational and skill-building program rooted in the cultural norms, values, and traditions of Hispanic populations. By grounding the program in findings from community focus groups, elements of cultural responsiveness, principles of trauma-informed practice, and evidence-based practices, the research team set out to develop a brief intervention to reduce mental health stigma and increase emotional well-being among Hispanic populations currently underserved by existing mental health service delivery models. The statistically significant change in stigma-related scores reflected in this study based on the effect size for SSOSH scores suggests a small-to-moderate effect of the intervention in reducing self-stigma related to seeking psychological help among participants retained in the analytic sample. This supports *Pensamientos y Pláticas* as a promising intervention to reduce self-stigma related to seeking psychological for Hispanic adults. Because this was an uncontrolled pilot study, the practical or clinical meaningfulness of this change should be examined in future studies using larger samples, comparison groups, and longer follow-up periods.

Although the present study did not observe a statistically significant increase in emotional well-being from participation in the program, various explanations warrant consideration in interpreting the findings. Engaging in mental health conversations may initially increase participants’ awareness of emotional distress, therefore bringing emotions to the surface. Additionally, the relatively short duration of the intervention may have been insufficient to capture meaningful changes in emotional well-being, or it may be that stigma-related attitudes change before measurable differences in emotional well-being. The program’s statistically significant impact on stigma reduction suggests it is a promising intervention at normalizing conversations around mental health and reducing self-stigma related to help-seeking and reflects a valuable stage in the process of supporting individuals in better understanding their mental health experiences and how to cope with mental health challenges. However, the intervention must be tested in a larger controlled study with longer follow-up to determine the impact of change in self-stigma on actual help-seeking behaviors and treatment utilization.

### Strengths and Limitations

The findings of this study should be interpreted with caution considering its strengths and limitations. The study’s strengths include engaging the community in the development and implementation of the program, grounding it in culturally relevant elements, and delivering it in settings where the primary focus is not on treating mental health conditions. Collectively, these strategies aimed to reach a population that may be less likely to seek mental health treatment related to self-stigma, but that may be experiencing unresolved mental health conditions at disproportionately higher rates when compared to populations more likely to seek and receive treatment. By collaborating with community partners providing services related to food security, community engagement, domestic violence, parenting, and overall community wellness, the study reached a diverse sample of participants, including individuals more likely to be experiencing mental health challenges alongside other challenges in meeting basic needs.

Despite these strengths, this study has several limitations. Given the significant variation in the number of modules completed, additional steps are needed to identify strategies to promote retention and attendance at all sessions. Despite this limitation, participant retention remained strong, with nearly three-quarters of participants included in the study’s analytic sample completing at least three out of the four modules while just under half completed all four modules. The research team views these completion rates as a success given the other challenges and demands study participants experience. Additional limitations stem from the research methodology. The use of convenience sampling limits the extent to which the sample can be considered representative of the broader population, thereby limiting the generalizability of our findings. The small sample was predominantly female and recruited through convenience sampling from specific community settings in the Paso del Norte region. These factors further limit the extent to which the findings can be generalized to broader Hispanic populations, including men, younger adults, individuals from other Latino subgroups, and Hispanic communities outside the U.S.–Mexico border region. Additionally, the absence of a comparison or control group limits the ability to attribute observed changes in participants to the intervention. Without a longitudinal design, the study is unable to measure the longer-term impacts of stigma reduction on seeking and engaging in mental healthcare, including help-seeking behaviors, future treatment engagement, and duration of service use. Nevertheless, preliminary findings suggest that *Pensamientos y Pláticas* shows promise as an intervention for mental health stigma reduction for Hispanic adults. Future research is needed to further enhance the evidence base for this intervention. Specifically, employing controlled research conditions (e.g., a comparison or control group) and using a longitudinal design would help assess the program’s effectiveness and actual impacts on help-seeking behavior.

## 8. Conclusions

For Hispanic adults, existing strategies for increasing mental health equity have fallen short of bridging the longstanding gap in help-seeking and accessing mental health services experienced by this population. As detailed in this article, the authors set out to develop an innovative and community-grounded mental health engagement program to help reduce mental health stigma and increase emotional well-being for Hispanic populations specifically. Initial findings suggest the program’s potential benefit at reducing mental health stigma. Such findings suggest that, rather than adapting existing interventions to improve cultural fitness to this population, new strategies are warranted.

*Pensamientos y Pláticas* was developed based on community recommendations for increasing mental health equity for an underserved population in an under-resourced region. By centering community knowledge, the program reflects shared community perceptions of need, as well as culturally relevant potential solutions for addressing these needs. The resulting community–academic partnership reflects an authentic effort to uplift the voices of community members, create space for connection-building, and enhance participants’ ability to cope with mental health challenges they may be experiencing. The impact of *Pensamientos y Pláticas* is further evident in group members exchanging phone numbers and taking pictures at the end of the program, as well as in requests for a “part two” to continue these conversations. Normalizing conversations around mental health, coupled with providing education and skill-building through a culturally responsive lens, represents a critical pathway towards advancing mental health equity for Hispanic populations.

## Data Availability

The participants of this study did not give written consent for their data to be shared publicly, so supporting data is not available. The program facilitator manual and participant workbooks are available upon request to the corresponding author.

## Supplementary Materials

The following supporting information can be downloaded at https://www.mdpi.com/article/10.3390/healthcare14152240/s1, Table S1: Participant Characteristics; Table S2: Paired-Samples *t*-Tests for Pre- and Post-Measures.
