# Supplementary material for: Preliminary Evidence of Feasibility, Acceptability, and Potential Benefit of a Culturally Responsive Mental Health Engagement Program for Hispanic Adults, Pensamientos y Pláticas
Source: Healthcare (Basel). 2026 Jul 23;14(15):2240. doi: 10.3390/healthcare14152240 (PMC13464743; doi:10.3390/healthcare14152240)
Supplement: Supplementary file 1 [file healthcare-14-02240-s001.zip › healthcare-4349327-supplementary.pdf]

Preliminary Evidence of Feasibility, Acceptability, and Potential Benefit of a Culturally Responsive Mental Health Engagement Program for Hispanic Adults, *Pensamientos y Pláticas*

Supplementary Materials

Table S1: Participant Characteristics.

Table S1

Participant Characteristics

Sociodemographic Data	*M (SD)*	*N*	%
Gender			
Female		38	97.4
No Response		1	2.6
Age (N = 37)	50.6 (12.9)		
Race & Ethnicity			
Hispana		9	23.1
Latina		5	12.8
Mexicana		5	12.8
Hispanic		3	7.7
Hispano		3	7.7
Black		2	5.1
Blanca		2	5.1
Black/Mexican/Italian		1	2.6
Cuban/American		1	2.6
Hispana/Latina		1	2.6
Hispana/Mexicana		1	2.6
Latina/Boliviana		1	2.6
Latina/Hispana		1	2.6
No Response		4	10.3
Marital Status			
Single		13	33.3
Married		12	30.8
Separated		4	10.3
Divorced		4	10.3
Widowed		3	7.7
Other		2	5.1
No Response		1	2.6
Education Level			
No School		2	5.1
Elementary School		5	12.8
Middle School		3	7.7

High School	4	10.3
GED/High School Equivalent	6	15.4
Technical College	4	10.3
Some College	2	5.1
Bachelor's Degree	8	20.5
Master's Degree	2	5.1
No Response	3	7.7
Annual Income Level		
$0	8	20.5
$1 to $9,999	6	15.4
$10,000 to $24,999	11	28.2
$25,000 to $49,999	5	12.8
$50,000 to $74,999	1	2.6
No Response	8	20.5
Previous Use of Mutual Aid/Self-Help		
Yes	12	30.8
No	21	53.8
No Response	6	15.8
Need for Mental Health Services		
Yes	20	51.3
No	13	33.6
No Response	6	15.8
Previous Use of Mental Health Services		
Yes	18	46.2
No	15	38.5
No response	6	15.8

Note. M = mean; *SD* = standard deviation.

Table S2: Paired-Samples t-Tests for Pre- and Post-Measures.

Table S2

Paired-Samples t-Tests for Pre- and Post-Measures

Measure	M_{pre}	M_{post}	SD_{pre}	SD_{post}	t	p	Cohen's d
WEMWB-Total	52.41	55.79	11.5	11.34	−1.94	0.60	−0.31
SSOSH	3.04	2.71	0.86	0.81	2.22	0.03	0.36

Note. M = mean; *SD* = standard deviation. Negative *t* values indicate higher post-scores relative to pre-scores.
